# Quaternion-based recurrent neural networks and machine learning for 3D modeling of superficial femoral artery dynamics: a theoretical framework for enhanced treatment outcomes

**DOI:** 10.3389/fsurg.2026.1896488

**Published:** 2026-08-06

**Authors:** Nikolaos D. Patelis, Eleni P. Georgiadi, Bahaa Arefai

**Affiliations:** 1 Mouwasat Hospital Riyadh, Riyadh, Saudi Arabia; 2 2nd Department of Vascular Surgery, Laiko Hospital, National & Kapodistrian University of Athens, Athens, Greece; 3 2nd Department of General Surgery, General Hospital of Nikaia “Agios Panteleimon”, Piraeus, Greece; 4 Hospital Universitari de Tarragona Joan XXIII, Tarragona, Spain; 5 Eastern Health Cluster, Dammam, Saudi Arabia

**Keywords:** 3D vascular modeling, machine learning, quaternion neural networks, restenosis prediction, superficial femoral artery

## Abstract

The superficial femoral artery (SFA) is highly susceptible to atherosclerotic disease, with restenosis rates which are exceeding 30%–50% following endovascular intervention. Current diagnostic approaches including two-dimensional angiography and duplex ultrasound are failing to capture the complex three-dimensional (3D) spatiotemporal dynamics of vessel remodeling during physiological motion. This theoretical framework is integrating quaternion-based recurrent neural networks (QRNNs) with machine learning pipelines to model dynamic 3D changes in SFA geometry from serial imaging data or continuous recording of SFA quaternion dynamics during activities by wearable *Internet of Things* devices. Quaternions are providing a singularity-free representation of 3D rotations, while QRNNs are processing quaternion-valued sequences through Hamilton product operations. The integrated pipeline is combining 3D convolutional neural network encoders for feature extraction, QRNN cores for temporal sequence modeling, and conditional generative adversarial networks for synthetic geometry generation. A multi-objective loss function is balancing quaternion prediction accuracy, geometric feature errors, geometric realism, and probability calibration. The framework is theoretically enabling predictive 3D modeling of vessel remodeling, simulation-driven personalized intervention planning, and quantitative risk stratification for restenosis. By grounding the approach in rigorous quaternion mathematics, QRNNs could achieve improved fidelity in capturing dynamic vessel behavior compared to Euclidean approximations. This theoretical framework may be able to address critical gaps in current treatment approaches and is demonstrating a potential for transforming SFA 3D analysis and peripheral artery disease management, once it has been tested and validated.

## Introduction

Peripheral artery disease (PAD) is affecting over 200 million individuals globally, with the Superficial Femoral Artery (SFA) representing the most frequently diseased arterial segment (1). The SFA's vulnerability is coming from its anatomical length, continuous biomechanical stress during hip and knee flexion, and hemodynamic complexity (2). Endovascular interventions including balloon angioplasty, stent placement, and atherectomy are the primary treatment modalities (3).

Despite technological advances, restenosis is occurring in 30%–50% of patients within 12 months post-intervention (4). Current diagnostic approaches, including two-dimensional angiography and duplex ultrasound, are providing limited spatial information and they are lacking temporal modeling (5). While CTA and MRA are offering superior 3D visualization, they are capturing static snapshots rather than dynamic vessel evolution (6).

The SFA's complex 3D behaviour during physiological movement cannot be adequately represented using the standard recurrent neural network architectures (7). Quaternions are providing mathematically elegant and computationally efficient representations of 3D rotations without singularities (8). Quaternion-based recurrent neural networks (QRNNs) are extending this advantage to sequential processing, enabling the modeling of temporal changes in vessel orientation and geometry (9).

This paper is presenting a theoretical framework which integrates QRNNs with machine learning to Internet-of-Things (IoT) data capture, analyze, and predict 3D SFA dynamics. This theoretical approach is aiming to eventually enable: (1) predictive 3D modeling of vessel remodeling, (2) simulation-driven treatment planning, (3) personalized intervention selection, and (4) quantitative risk stratification for restenosis.

## Theoretical foundations: representing 3D vessel dynamics

### Quaternion encoding for arterial geometry

Accurate representation of 3D vessel structure is requiring encoding both position and orientation in space. The SFA centerline can be parameterized along arc-length *s* using the Frenet-Serret frame, which comprises position vector r(s), tangent vector t(s), normal vector n(s), and binormal vector b(s) (10). Each frame's orientation at time *t* is mathematically encoded as a unit quaternion:

$$q(s,t)=\cos\left(\frac{\theta }{2}\right)+usin\left(\frac{\theta }{2}\right)$$

where θ is representing the rotation angle and u denotes the normalized rotation axis. This quaternion representation is theoretically superior to Euler angles because it is avoiding singularities and is providing continuous interpolation (11). Complementing the quaternion-encoded orientation, local geometric features are captured as scalar quantities: formal Frenet-Serret curvature $\begin{array}{c} \kappa (s,t)=\frac{\partial t(s,t)}{\partial s}=\sqrt{\underset{i\in \{x,y,z\}}{\sum }{\left(\frac{\partial^{2}i}{\partial s^{2}}\right)}^{2}} \end{array}$ and luminal diameter d(s,t). These scalar features are quantifying the degree of vessel twisting and stenosis severity, respectively.

The complete feature vector at each centerline point is becoming f(s,t)=[q(s,t),κ(s,t),d(s,t)], integrating rotational and dimensional information. This hybrid representation is particularly advantageous for the SFA, which is undergoing complex 3D deformation during normal physiological motion. Serial imaging acquisitions at discrete time points $t_{1},t_{2},\ldots ,t_{T}$ are producing feature sequences $F_{t}=[f_{1}(t),\ldots ,f_{N}(t)]$, capturing temporal evolution of vessel morphology which is driven by progressive atherosclerotic remodeling and post-intervention neointimal proliferation.

### Advantages over traditional representations

Euclidean coordinate systems are inherently distorting 3D rotations, leading to accumulated errors in sequential prediction tasks (12). Standard neural network architectures are processing feature vectors as generic high-dimensional points, failing to respect the geometric constraints of 3D space. In contrast, quaternion representations are enforcing rotational geometry at the architectural level, reducing dimensionality (4-element vectors vs. 9-element rotation matrices) while improving numerical stability (13). For vascular applications, this is translating to more accurate predictions of dynamic orientation changes which are occurring during gait and physiological loading.

## Quaternion recurrent neural network architecture

### Core architectural design

The QRNN is processing quaternion-valued sequences through explicit Hamilton product expansion, the quaternion analog of matrix multiplication. The fundamental recurrence relation is:

$$\begin{array}{rl} & q_{1}⊗q_{2}=(a_{1}a_{2}-b_{1}b_{2}-c_{1}c_{2}-d_{1}d_{2})+\ldots \\ & +\;(a_{1}d_{2}+b_{1}c_{2}-c_{1}b_{2}+d_{1}a_{2})k \end{array}$$

where ⊗ is denoting the Hamilton product, $h_{k}$ is representing the quaternion-valued hidden state, W and U are learnable quaternion weight matrices (parameterized as 4×4 real matrices for computational efficiency), and σ is a quaternion activation function (e.g., component-wise hyperbolic tangent followed by quaternion normalization to maintain unit length) (14).

The architecture is typically comprising 2–3 stacked QRNN layers with 128–256 hidden units per layer, progressively extracting hierarchical representations of vessel dynamics. Attention mechanisms can be incorporated to selectively weight distant time steps, focusing computational resources on critical phases such as post-treatment remodeling periods (15). The final QRNN layer is outputting predictions for future states, including predicted orientation changes Δq, curvature changes Δκ, and diameter evolution Δd.

### Theoretical advantages for vascular modeling

QRNNs are inheriting fundamental advantages from quaternion mathematics and recurrent architectures: (1) Rotational fidelity: Hamilton product operations are respecting 3D geometric constraints, reducing prediction errors in dynamic scenarios; (2) Parameter efficiency: Quaternion-based weight sharing is reducing model size compared to equivalent real-valued networks operating on flattened rotation matrices; (3) Non-Euclidean geometry: The framework is naturally modeling the non-Euclidean manifold of 3D rotations, theoretically improving long-term forecasting accuracy. These properties are particularly important for the SFA, where complex 3D deformation during normal activity is driving pathophysiological changes.

## Integrated machine learning pipeline

### End-to-end framework design

The QRNN is forming the temporal core within a comprehensive machine learning pipeline architecture which is designed to integrate multiple computational stages. A 3D convolutional neural network (CNN) encoder (e.g., 3D ResNet variants) is processing volumetric imaging data acquired from CTA or MRA modalities, performing automated SFA segmentation through fully convolutional approaches and extracting initial centerline representations which are converted to quaternion sequences. The encoder is learning hierarchical feature representations that capture vessel morphology at multiple scales, from fine vessel wall details to large-scale tortuosity patterns. The QRNN is then processing these quaternion sequences, generating temporal predictions based on learned spatiotemporal dependencies from the training cohort. A decoder module—potentially incorporating conditional generative adversarial networks—is synthesizing hypothetical 3D vessel geometries which are conditioned on QRNN outputs and treatment parameters including stent diameter, material properties, coating composition, lesion calcification burden, and vessel wall compliance characteristics. Finally, a risk assessment head is computing clinical outputs, particularly restenosis probability *P_resten_(s,t)* at each vessel location and time point, along with confidence intervals quantifying prediction uncertainty.

The pipeline architecture is incorporating several feedback loops and refinement mechanisms. A validation module is comparing synthesized geometries against observed follow-up imaging to iteratively improve model calibration. Feature importance analysis is being performed to identify which vessel characteristics and treatment parameters are most strongly influencing restenosis outcomes. The modular design is allowing for selective updating of individual components—for instance, the CNN encoder can be retrained on new imaging datasets without requiring retraining of the QRNN core or risk assessment head.

### Loss function and training strategy

The integrated loss function is balancing multiple competing objectives across different prediction tasks:

$$\begin{array}{c} L_{geo}=1-{\left(a_{p}a_{g}+b_{p}b_{g}+c_{p}c_{g}+d_{p}d_{g}\right)}^{2} \end{array}$$

The first term is enforcing quaternion prediction accuracy through squared error minimization, which is appropriate because quaternion space is already normalized. The second term is penalizing geometric feature errors (curvature and diameter) using SmoothL1 loss, which is providing robustness to outliers compared to pure squared error. The third adversarial term is ensuring that simulated geometries are realistic and indistinguishable from authentic vessel morphologies generated through biomechanical processes. The fourth calibration term is aligning predicted restenosis probabilities with observed clinical outcomes through techniques such as expected calibration error minimization, ensuring that when the model is predicting 30% risk, approximately 30% of such cases are experiencing restenosis.

Theoretically, this multi-objective formulation is capturing both the precision which is required for orientation prediction and the quality which is needed for clinically plausible simulations. Hyperparameter tuning of the weighting coefficients *λ*_1_ through *λ*_4_ is being performed through Bayesian optimization on a held-out validation set.

Training would employ supervised learning on paired pre/post-intervention imaging data, with synthetic examples which are generated through finite element analysis simulations or biomechanical models of arterial remodeling. Self-supervised pretraining on unlabeled imaging sequences could enhance robustness and reduce data requirements, using quaternion autoencoders to learn vessel-specific representations before supervised fine-tuning on restenosis prediction tasks. Data augmentation strategies including temporal interpolation, geometric transformations, and addition of synthetic noise are being employed to improve generalization.

### Internet of things-based quaternion tracking of SFA segments

Beyond static imaging-based analysis, continuous monitoring of SFA quaternion dynamics during daily activities is requiring integration with wearable IoT devices that are providing longitudinal behavioral context. This IoT-based approach has already been tested and used in athletes, providing 3D spatial and temporal data (16–23). Miniaturized inertial measurement units (IMUs) which are equipped with triaxial accelerometers, gyroscopes, and magnetometers can be embedded in thigh-mounted wearable patches which are positioned at defined anatomical landmarks corresponding to SFA segment boundaries (20, 21). These IMUs are directly measuring 3D rotational velocities and accelerations of limb segments, which can be transformed into quaternion representations of local leg orientation through kinematic calculations. Through sensor fusion algorithms (e.g., complementary filters or extended Kalman filters), quaternion estimates from multiple distributed IMUs are integrated to reconstruct continuous 3D orientation sequences q_IoT_(t) throughout the gait cycle and daily activities, capturing both structured walking patterns and spontaneous free-living movements.

These quaternion time-series, which are transmitted wirelessly via Bluetooth® Low Energy (Bluetooth Special Interest Group, Kirkland, WA, USA) to a mobile or cloud computing platform, are providing continuous input to QRNN models which are trained on imaging data, enabling real-time prediction of vessel stress states and restenosis risk based on biomechanical loading patterns. The wearable sensors are also measuring movement quality metrics including gait symmetry, cadence variability, and activity intensity, which are providing additional predictive features beyond pure orientation data. The integration of IoT-derived quaternion trajectories with hospital-acquired imaging quaternions is creating a longitudinal digital phenotype, bridging episodic imaging snapshots with continuous behavioral monitoring and potentially identifying activity patterns which are precipitating adverse vascular remodeling. For example, high-intensity activities or repetitive twisting movements during certain daily tasks could trigger excessive vessel stress, which the QRNN model could detect and flag for clinical attention. The foundational framework of employing Bluetooth IoT wearables for the continuous transmission of spatial and inertial movement metrics has been extensively described and validated (22, 23).

## Clinical applications and problem-solving potential

The proposed framework is addressing multiple critical gaps in current SFA management and enabling clinically actionable predictions:

### Predictive 3D modeling

By forecasting vessel evolution over months to years based on initial post-intervention geometry and patient biomechanical characteristics, clinicians could anticipate complications such as stent fracture from excessive tortuosity development, excessive neointimal proliferation in high-curvature regions, or premature stent strut fracture from repetitive mechanical fatigue (24, 25). This predictive capability would enable preemptive device selection, prophylactic re-intervention before symptoms develops, or intensified medical therapy in high-risk cases. The model could identify which anatomical regions of the SFA are most vulnerable to adverse remodeling for a given patient's biomechanical loading patterns, allowing targeted therapeutic interventions.

### Personalized intervention planning

Integration of patient-specific biomechanical factors (gait patterns, limb loading magnitudes, activity levels, occupational demands) is allowing simulation of intervention outcomes before proceeding to actual treatment. Multiple competing intervention strategies could be simulated computationally to predict long-term outcomes, potentially identifying high-risk scenarios where standard stenting approaches are predicted to fail, and alternative approaches such as atherectomy, drug-eluting balloons, or bypass surgery would be preferable. Once validated and clinically tested, this simulation-driven planning could potentially reduce the burden of repeat interventions and improve long-term patency rates by selecting the optimal initial strategy for each patient.

### Risk stratification

Computational risk heatmaps along the vessel centerline are identifying focal regions of highest restenosis probability based on local geometric and biomechanical factors, informing treatment intensity and follow-up surveillance strategies. Rather than applying uniform surveillance protocols to all patients, the framework could enable risk-stratified follow-up where high-risk zones are receiving more intensive monitoring while low-risk segments are requiring less frequent imaging. This approach could optimize healthcare resource utilization while improving outcomes through earlier detection of incipient restenosis in vulnerable regions.

### Treatment optimization

Comparative simulations of competing interventions (stenting vs. atherectomy, different stent designs, different stent materials, different sizing strategies) are providing quantitative outcome predictions supporting evidence-based selection. The framework could guide decisions regarding stent diameter, length, material composition, and drug payload based on patient-specific vessel characteristics and loading patterns. For example, the model could predict that a particular patient's SFA would benefit more from a flexible nitinol stent compared to stainless steel, or that a longer overlapping stent configuration would better resist excessive tortuosity development.

### Limitations

While promising, this framework lacks empirical validation—no datasets or benchmarks demonstrate QRNN usage superiority over Long Short-Term Memory networks (LSTMs) or standard RNNs on real SFA data. IMU-to-vessel mapping oversimplifies biomechanics and patient variability. Training requires large, paired imaging cohorts, which are scarce at present and should be collected prospectively. Computational demands (3D CNNs, QRNNs etc.) challenge clinical deployment due to high related costs. IoT wearables raise privacy concerns and patient consent is obligatory before data collection.

To address the lack of empirical evidence, a structured validation protocol was introduced, shifting the work from a theoretical hypothesis to a verifiable proof-of-concept.

We have formally acknowledged the theoretical nature of this study. The proposed validation protocol (Table 1), including benchmarking against LSTM/Gated Recurrent Units (GRUs) baselines with MAE and Dynamic Time Warping (DTW) metrics, serves as our roadmap for future implementation.

**Table 1:** Benchmark framework and validation roadmap.

Feature	Standard RNN	Proposed QRNN	Primary target performance metrics
Geometric manifold	Linear (*ℝ^n^*)	Hypersphere (S^3^)	Mean absolute error of 3D spatial coordinates
Rotational singularities	Present	None	Geodesic distance/Quaternion mean squared error
Temporal dynamic distortion	Distorts over long-horizon trajectories	Shape-invariant sequence alignment	Dynamic time warping alignment distance
Parameter overhead	High parameter density $O(N^{2})$	Highly compressed via Hamilton product $O\left(\frac{N^{2}}{4}\right)$	Total count of trainable weights & inference latency (ms)

### Detailed proof-of-concept validation protocol

To transition this framework from a theoretical hypothesis to an empirically verifiable paradigm, a structured three-phase validation execution plan is established:

Data Generation & Representation: Given the present scarcity of long-term paired clinical datasets, an initial benchmark testbed will be constructed by simulating 3D SFA centerline trajectories using mechanical Finite Element Analysis (FEA) across 200 distinct simulated gait configurations. Spatial centerlines will be mapped to continuous sequential Frenet-Serret frames, encoding localized 3D directional orientations into unit quaternion time-series vectors.

Comparative Training Execution: The QRNN will be trained directly alongside standard real-valued RNN, LSTM networks, and GRUs. Model training will employ an identical 5-fold cross-validation configuration (80% training, 20% validation split). Baselines will ingest flattened 12-dimensional Euclidean array representations, while the QRNN natively tracks structural quaternion features through internal Hamilton product algebra.

Statistical Verification Targets: Predictive fidelity will be quantified across multi-step forecasting horizons modeling vessel morphology at 3, 6, and 12 months post-intervention. Empirical superiority will be validated if the QRNN demonstrates a statistically significant (*p* < 0.05) reduction in DTW trajectory misalignment and geodesic tracking error over standard architectures, while simultaneously yielding a reduction of at least 50% in total model parameter footprint.

## Conclusion

This theoretical framework is demonstrating compelling potential for quaternion-based recurrent neural networks and machine learning to transform 3D vascular analysis. By grounding the approach in rigorous quaternion mathematics rather than *ad-hoc* Euclidean approximations, the framework could theoretically achieve superior fidelity in capturing dynamic 3D vessel behavior. The integration of IoT wearable sensors with hospital imaging is creating a comprehensive digital phenotype spanning from episodic clinical encounters to continuous real-world biomechanical monitoring. Current restenosis rates of 30%–50% are representing substantial patient harm and healthcare costs, therefore a predictive framework which can prevent even a fraction of these failures would deliver significant clinical and financial benefit. Future work should be prioritizing prospective validation studies, regulatory pathway clarification, and clinical adoption strategies to translate this theoretical framework into clinical practice.

## Data Availability

The original contributions presented in the study are included in the article/Supplementary Material, further inquiries can be directed to the corresponding author.
